# Characteristics of the cancer stem cell niche and therapeutic strategies

**DOI:** 10.1186/s13287-022-02904-1

**Published:** 2022-06-03

**Authors:** Feng Ju, Manar M. Atyah, Nellie Horstmann, Sheraz Gul, Razi Vago, Christiane J. Bruns, Yue Zhao, Qiong-Zhu Dong, Ning Ren

**Affiliations:** 1 General, Visceral and Cancer Surgery, University Hospital of Cologne, Kerpener Straße 62, Cologne, Germany; 2 Department of Liver Surgery and Transplantation, Liver Cancer Institute, Zhongshan Hospital, Fudan University, No. 180 Fenglin Road, Shanghai, 200032 China; 3 Key Laboratory of Carcinogenesis and Cancer Invasion of Ministry of Education, Shanghai, China; 4 Fraunhofer Institute for Translational Medicine and Pharmacology ITMP, 22525 Hamburg, Germany; 5 Fraunhofer Cluster of Excellence for Immune-Mediated Diseases CIMD, 22525 Hamburg, Germany; 6 Avram and Stella Goldstein-Goren, Department of Biotechnology Engineering, Ben-Gurion University of the Negev, Beer-Sheva, Israel; 7 Institute of Fudan-Minhang Academic Health System, Minhang Hospital, Fudan University, Shanghai, 201199 China; 8 Key Laboratory of Whole-Period Monitoring and Precise Intervention of Digestive Cancer of Shanghai Municipal Health Commission, Shanghai, 201199 China

**Keywords:** Cancer stem cells, Chemokines, Hypoxia, Metastasis, Niche/microenvironment

## Abstract

Distinct regions harboring cancer stem cells (CSCs) have been identified within the microenvironment of various tumors, and as in the case of their healthy counterparts, these anatomical regions are termed “niche.” Thus far, a large volume of studies have shown that CSC niches take part in the maintenance, regulation of renewal, differentiation and plasticity of CSCs. In this review, we summarize and discuss the latest findings regarding CSC niche morphology, physical terrain, main signaling pathways and interactions within them. The cellular and molecular components of CSCs also involve genetic and epigenetic modulations that mediate and support their maintenance, ultimately leading to cancer progression. It suggests that the crosstalk between CSCs and their niche plays an important role regarding therapy resistance and recurrence. In addition, we updated diverse therapeutic strategies in different cancers in basic research and clinical trials in this review. Understanding the complex heterogeneity of CSC niches is a necessary pre-requisite for designing superior therapeutic strategies to target CSC-specific factors and/or components of the CSC niche.

## Definitions and background

Stem cells are undifferentiated cells that have the capacity to self-renew and proliferate for longer than non-stem cells as well as having the ability to generate multiple types of cells in the body [1, 2]. Regardless of their proliferation potential, stem cells are usually quiescent, remaining in an inactive dormant state (G1, G0) and are protected from cellular damage or mutations [3, 4].

In general, stem cells exhibit various levels of differentiation potential, starting with totipotency (greatest differentiation potential), pluripotency, multipotency, oligopotency and finally unipotency/monopotency (stem cells can generate only one cell type) [4]. The survival of stem cells is ensured at two levels: cellular asymmetrical division and population asymmetrical renewal. With asymmetrical division, one stem cell gives rise to a stem cell and one differentiating cell. With population asymmetry, one stem cell produces two stem cells or two differentiating cells. As the frequency of these two processes are similar, both result in a comparable amount of stem cells and differentiating cells [5].

Some types of tissues have higher turnover and renewal rates than others (such as gastric and intestinal tissues and bone marrow as compared to brain or liver tissues), making the capability of stem cell self-renewal essential for their replenishment and providing an explanation for their broad presence in such tissues [6]. Many studies have explored whether the stemness of these cells is a result of an independent mechanism or the interaction with other cells and their surrounding environment and it has been established that these interactions are vital for the survival of stem cells [7].

Due to the inherent nature of stem cells, their use in regenerative medicine and stem cell-based therapy has been widely explored. However, to date, clinical applications of such therapeutic approaches are only available in hematopoietic malignancies and limited immune deficiencies [4].

Similar to normal stem cells, Cancer stem cells (CSCs) are also able to self-renew and differentiate into tumor cells [8]. With the multipotent capacity of CSCs, heterogeneous lineages of different kinds of cancer cells can be generated [9]. The ability of CSCs to initiate and reconstitute tumor lesions together with features such as differentiation and chemo/radiotherapy resistance has been widely investigated [10]. Although many features of CSCs are similar to normal stem cells, an important difference is that normal stem cells are usually dormant during adulthood until their regeneration ability is required, whereas this ability of CSCs is active. This leads to the possibility of detecting CSC based on markers found specifically in active stem cells but not dormant ones, such as PSF1 which has been identified in upregulated hematopoietic stem cells (HSCs) [7]. Many technologies and assays have been proposed for the identification of CSCs including microsphere assay, serial dilution assay, side population assay and aldehyde dehydrogenase activity assay, the gold standard for the CSC identification is the sphere colony formation assay and in vivo xeno transplantation of tumor cells into immunodeficient mouse models. Although each of these methods has limitations, they assist in identifying CSCs in several tumors such as breast cancer, brain cancer, liver cancer, stomach and colon cancer [10].

The fundamental mechanism by which CSCs function in cancer progression is unclear. A number of proposals have been made with regards to this, such as CSCs derived from normal somatic cells regain stemness due to cancerous changes and acquired genetic/epigenetic mutations [11]. For this, two models prevail, namely the hierarchical and stochastic models. In the hierarchical model, tumor initiation begins with stem cells that escape normal growth control and regulation. Therefore, they can transform into CSCs which give rise to a distinct population of cells forming the biological basis of tumors. This model has been validated clinically and would explain why only a full elimination of CSCs can prevent the relapse of cancers. Nevertheless, this model is unable to explain the interaction between CSCs and other differentiated cancer cells. In contrast, the stochastic model suggests that under suitable conditions all tumor cells (differentiated or not) can initiate further lesions. However, this model has limitations in explaining the relation between tumor heterogeneity and the capacity of initiating tumors. These findings have led to the concept of cellular plasticity, where both models are merged. Based on the genetic/epigenetic and microenvironmental signals, it is suggested that cancer cells have the ability to shift between stemness and differentiation states [12].

**Fig. 1:**
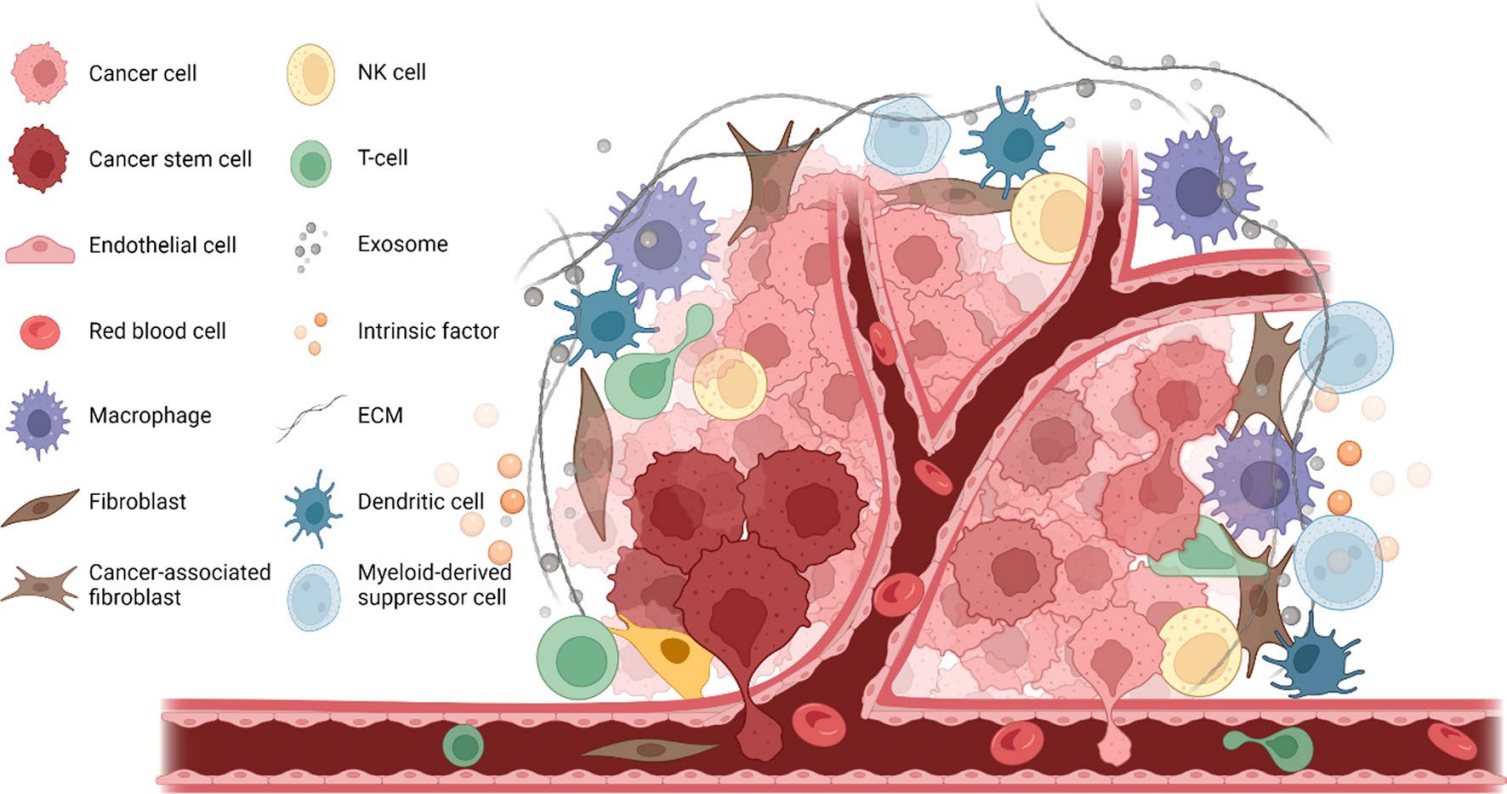
CSC niche in solid tumor. CSC niche has a complex microenvironment, it usually includes cancer cells, cancer stem cells, stromal cells, endothelial cells, fibroblasts, cancer-associated fibroblasts, ECM, exosomes and intrinsic factors. These components together contribute to the CSC renewal and maintain tumor malignancy

The microenvironment of CSCs is essential for their function and due to their complex nature and interactions with other components and factors, is referred to as the CSC niche. The CSC niche usually includes niche cells, such as cancer cells, stromal and endothelial cells extracellular matrix (ECM), signaling molecules, intrinsic factors, blood vessels and other cellular and acellular components such as exosomes. The components and structure of the niche can vary among organisms and different types of tissues in order to provide distinct functions in response to the needs of the tumor (Fig. 1) [13–16]. CSC niches have been identified in many locations including intestinal, tissues and neural tissues, aiding the regulation and maintenance of stem cell renewal and differentiation [6, 17].

Due to the rarity of stem cells (compared to non-stem cells), it is challenging to clearly elucidate the mechanisms in the CSC niche [1]; however, some advances have been made with regards to this. The regulation mechanisms in the niche includes intrinsic mechanisms (associated with transcription factors expressed by cells), and extrinsic mechanisms (based on the signaling of the microenvironment and the connection to ECM). Disruption or interference in these mechanisms can lead to phenotypic changes that alter homeostasis of the niche [18]. Since the niche can contain more than one type of stem cells, competition between different stem cells within the niche is considered to control its function by factors such as E-cadherin [19], where the strength of the stem cell-niche connection can directly affect the fate of stem cells [14]. Even when only one type of stem cells are present in a niche, they still may lose their connection and, therefore, be replaced due to limited space or competition for occupancy [5] and this concept is crucial with regards to CSC niches.

Another important CSC feature is their ability to change and modify nearby stroma by CSC secreted proteins and molecular components, such as ECM proteins. The mechanism of ECM regulation has already been identified in different mammalian stem cells. In return, the niche can effectively regulate the biochemical status of keeping CSC in dormant. This is essential for the fate and plasticity of CSC and plays a role in their resistance to conventional therapies [15]. In order to better understand the characteristics of the CSC niche, we summarized the molecular features and mechanisms of the niche and reviewed the different therapeutic strategies targeting the CSC niche in various cancers from both basic and clinical aspects.

## Conditions of the CSC niche

### Hypoxia and its role in CSCs

Hypoxia is known for its role in the maintenance of the undifferentiated status of CSCs. The location of CSCs in hypoxic niches allows them to easily keep their slow cell cycling status with reduced proliferation, compared to a cycle with normal levels of oxygen. It is suggested that the rapid expansion of tumor mass and its vascular supply system might be the reason for tumor hypoxia that is responsible for the activation of several signaling pathways required in CSC functions. In solid tumors, the rapid growth of tumor tissues leads to a decrease in the quality of vascularization which affects the perfusion of blood and diffusion of substances it carries. That can be a cause for the poor outcome and the resistance of hypoxic niches against chemotherapy [20]. The hypoxic state of tumors is also linked to therapeutic resistance and local invasion [21, 22]. It has also been shown that HSCs under hypoxic conditions can enhance the stemness of HSCs and shift cells from a quiescence to renewal phase [23]. Low levels of oxygen can also protect cells from DNA damage caused by oxidative stress which are common in aerobic metabolism. Studies have shown that rates of cellular damage are significantly higher at 20% O_2_ relative to 3% O_2_. Low oxygen tension of 1% has also been shown to maintain the pluripotency of stem cells; therefore, hypoxia is essential for maintaining CSC characteristics [16].

In contrast to aerobic environments, hypoxic tissues use anaerobic glycolysis to metabolize glucose into lactate which produces much less ATP than in normal metabolism. This explains why cancer cells use higher rates of glucose compared to normal cells in aerobic conditions. However, reduced mitochondrial function is required, which is one way in which metabolic needs are down regulated in cancer cells and hypoxic niches. Such a difference between cancer cells and normal cells is usually controlled by the changes of oncogenes and tumor suppressor mutation profiles [20].

**Fig. 2:**
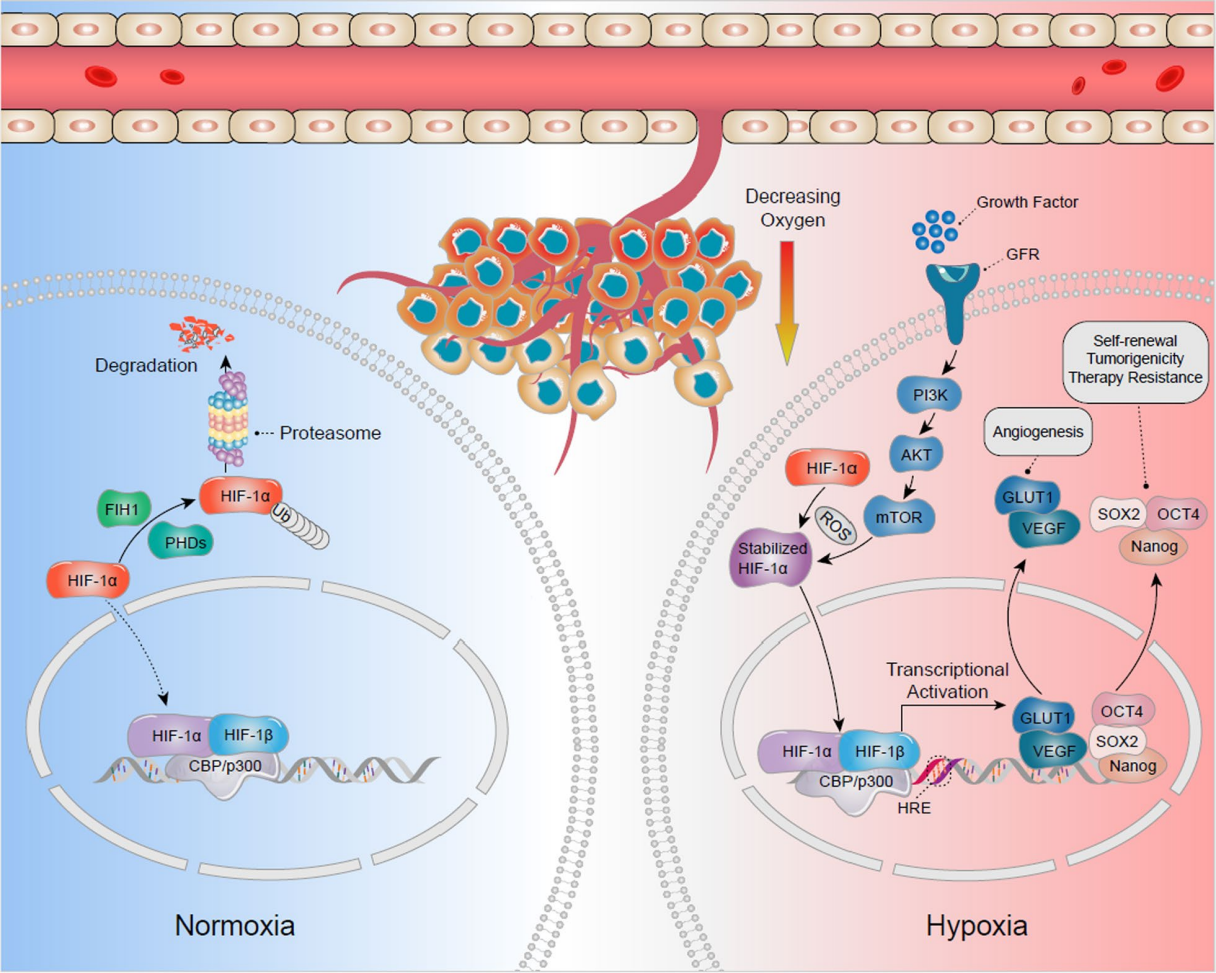
The hypoxic cancer stem cells (CSC) niches on CSC regulation. HIF-1α plays an important role in hypoxic process. Under normoxic conditions, HIF-1α is hydroxylated and inactivated by the function of poly1 hydroxylase domain-containing enzymes (PHDs) and Factor Inhibiting HIF (FIH1), and finally results in degradation. Under hypoxic conditions, both PHDs and FIH1 are inactivated, and HIF-1α stabilizes with the function of Reactive Oxygen Spices (ROS). HIF-1α could also be activated via activation of the PI-3 kinase/Akt-signaling pathway, which is induced by growth factor signaling. The stabilized HIF-1α translocates from cytoplasm to nucleus, where it dimerizes with HIF-1β and bind to Hypoxia Response Element (HRE). The heterodimer activates the downstream target genes transcription at these sites upon cofactor (CBP/p300) recruitment. These target genes in hypoxic niche, including Vascular Endothelial Growth Factor (VEGF), Glucose Transporter 1 (GLUT1), SOX2, Nanog and Octamer-binding Transcription Factor 4 (OCT4), may determine the fate of CSCs such as angiogenesis, self-renewal, tumorigenicity and induced therapy resistance

Many elements and factors participate in the control of hypoxic niches and related pathways such as mammalian target of rapamycin (mTOR) and the endoplasmic reticulum (ER) stress response. Examples of important transcription factors involved in the niche mechanisms are hypoxia inducible transcription factors (HIFs). Belonging to bHLH–PAS family of transcriptional factors, HIFs regulate many genes and play a role in oxygen homeostasis, glucose and iron metabolism and erythropoiesis [24–26]. These factors are also considered to be involved in several mechanisms such as the survival of cells under hypoxic conditions, cellular transcriptional responses, and activation of several signaling pathways. For example, expression of HIF-1α, CSC can promote tumor progression and metastasis. This transcription factor also affects genes involved in the regulation of the Notch and Oct4 pathways, as well as genes responsible for angiogenesis such as VEGF and GLUT-1 [1] by binding to HIF-1β and translocating to the nucleus where it can activate gene transcription. HIF signaling pathway can activate the angiogenic switch during tumor progression which is needed for the maintenance of oxygen homeostasis during the growth of tumor mass [27] (Fig. 2).

HIFs also play a role in regulating CSC proliferation, self-renewal, and tumorigenicity. The surface markers of CSC such as CD133 and CD44 show increased expression in hypoxic conditions [28, 29]. With regards to therapy response, radiation has been found to double HIF-1 activity within 24–48 h following exposure, imposing further limitations on the outcome of such therapeutic approaches in cancer patients. Even after HIF activity is lost (usually occurring within minutes of activation), the targets may still show certain activities [20].

### Metastasis of the CSC

Metastasis of CSCs was originally studied in hematopoietic malignancies followed by solid tumors which exhibited a similar phenomenon in brain, colon, breast, skin and pancreas cancers [30–34]. Most of these cancers are associated with specific metastasis-related genes as well as epigenetic amplification of cellular survival and renewal mechanisms than depend on driver mutations. Although migration occurs at an early stage of tumor progression, metastasis appears to take longer, due to the fact that not every migrated cell can initiate a tumor in distant locations and form a metastatic colony. Many conditions and mechanisms are required in both the migrated cells and the novel home tissues for this to occur, for example migrated cells must overcome many obstacles including lethal signals from reactive stroma that can downregulate the anti-apoptotic pathways in CSCs during the journey to the new environment [35]. CSCs must also survive the lack of growth factors and other environmental factors that are used to support their stemness and regulate their proliferation in the original niche [12, 36, 37]. Finally, after reaching the new site, the survivor CSCs need to induce specific niche factors to modulate the new niche according to their requirements.

It has also been found that one of the important stromal factors is POSTN (periostin) that normally plays a role in extracellular matrix formation and the development and function of teeth, bones, and heart tissues. In primary lesions, fibroblasts are responsible for POSTN expression; therefore, migrated tumor cells have to induce this factor in secondary lesions in order to successfully infiltrate into the new organs. POSTN is therefore a potential target for anti-metastasis therapies as it is considered to play a role in cancer stem cell maintenance. However, its deficiency only affects metastasis of tumors, but not normal cells nor primary lesions. Therefore, combination therapies may be necessary, but targeting POSTN alone may still prevent metastasis [38].

Following invasion of a new site, CSCs form a new niche to ensure their survival in the new environment where they face an aggressive immune response and a lack of survival signals and supportive stroma. Although most organs and tissues fend off CSC metastasis, some of these target tissues may be more susceptible than others. This creates an opportunity for CSCs to modulate the surrounding environment and build the metastatic niche (a novel niche in the new site that contains CSCs, niche components produced by CSCs themselves, and components produced by surrounding stromal cells) [12, 35].

### Epithelial to mesenchymal transition

**Fig. 3:**
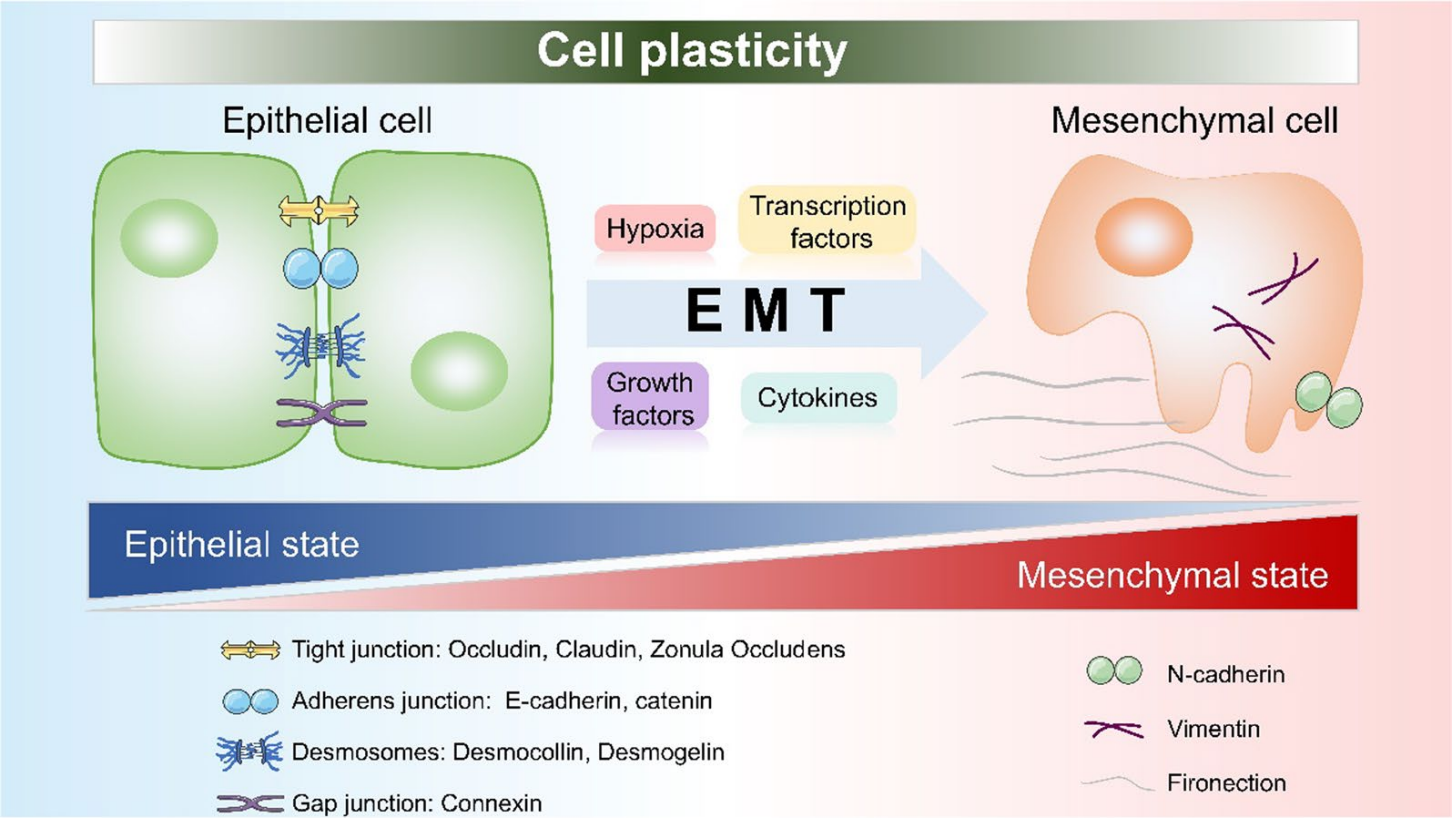
EMT and CSC niche. The crucial events in EMT are the dissolution of the epithelial cell–cell junctions, which include tight junction, adherens junction, desmosomes and gap junction. In CSC niche, those junctions start to break down via the induction of hypoxia, transcription factors, growth factors and cytokines. Then, the cells switch to a spindle-shaped mesenchymal morphology, as well as express markers (N-cadherin, vimentin and fibronectin) which would help to maintain the mesenchymal cell state

During the development of tissues, some epithelial cells exhibit changes in their characteristics (such as a loss of adhesion contact with other cells and cellular polarity) and transform into a mesenchymal type with improved ability to relocate into different tissues. This phenomenon is termed the epithelial to mesenchymal transition (EMT) [1]. In cancer niches, transcription factors (e.g., Snail, Twist, Six1, Slug, Cripto, Zeb 1–2, E12-46, and others) can affect the connection of cells by activating EMT and downregulating important molecules such as E-cadherin and catenins. These molecules play an essential role in cellular adherens junctions (AJs) [9]. EMT is important during the development of organisms due to its impact on tissue morphogenesis; however, it can become a cause of concern in cancer tissues due to the risk of metastasis. In addition, cells having undergone EMT are associated with stronger invasiveness and resistance against apoptosis. They usually acquire features similar to CSCs and recent studies to suggest that the CSC itself may be a result of EMT (since all signals and conditions needed to initiate EMT can come from tumor microenvironment) [15]. It has also been reported that the de-differentiation of cancer cells into CSCs occurs after those cells have gone through EMT and migrate to other locations, thus indicating the important role of EMT in this process [39]. The interactions within the niche between microenvironment cells and components can also initiate EMT, with participation of cytokines and growth factors. Such interactions were also found to play a role in maintaining stemness of CSCs in several cancers such as breast and oral squamous cancers [15] (Fig. 3).

Additional studies found that EMT indeed strongly correlates with hypoxia, with HIF-1α activating several EMT-related transcription factors such as Snail and Twist. Hypoxia can also elevate the CSC self-renewal genes expression levels and activate related signaling pathways such as Wnt and Notch [40, 41]. By activating EMT, hypoxic niches begin a chain of steps that eventually lead to metastasis, which also includes extracellular matrix modulation, intravasation, circulation, extravasation, homing, pre-metastatic niche formation, and mesenchymal epithelial transition (MET) [20].

## Molecular mechanisms in the CSC niche

### Essential molecules in the niche

The homeostasis of the CSC niche is based on the interaction between different cells and components of the microenvironment. In order to maintain this homeostasis, many complicated mechanisms and molecules are required. Some of these molecules have been mentioned already in this review. In this section, we discuss other important molecules in the CSC niche modulation such as chemokines, cytokines and adhesion molecules.

Chemokines are common components in niche interactions, with one of the important chemokines discovered being SDF-1a and its receptor CXCR4. Studies in HSC found CXCR4 as essential for the retention, maintenance, and homing of tumor cells. The receptor is also important for the regulation of the CSCs’ renewal capacity. Mesenchymal stem cells (MSCs) are considered as a major source of the SDF-1 production whose expression is linked to metastasis in solid tumors (e.g., breast, liver, and lung cancers). Therefore, this chemokine has been suggested to play a contributing role in the changes that migrated CSCs apply on the surrounding environment during the initiation of metastasis colonization [1, 6, 15]. Another important component is reactive oxygen species (ROS) which usually affects the hypoxic conditions of the cancer niche in a dynamic manner. This can lead to DNA damage and changes in protein expression which are required in DNA repair [20]. Altering ROS levels in the cell also affects cellular stress responses which may favor tumor cells over normal ones. By increasing the levels of several factors (e.g., HIF-1α, HGF, EGF, TGF-β, TNF-α, TPA, MMP-3, and even micro-RNA), ROS can enhance and help the EMT and promote the metastasis of tumor cells [42].

Another family of molecules required for CSC metastasis is the cytokine family. CSCs, together with other tumor cells, can secrete different cytokines in the niche. Such cytokines and chemokines can further recruit several types of immune cells to create immunosuppression (e.g., tumor-associated neutrophils (TAN), tumor-associated macrophages (TAM), and myeloid-derived suppressor cells (MDSC)). Other cells involved in the niche region and are affected by CSCs include cancer-associated fibroblasts (CAFs), which are known to increase proliferation, enhance ECM production and secret essential factors such as CXCL12, VEGF, PDGF, and HGF [12].

A crucial ability of stem cell niches is the self-renewal of cells and cell–cell adhesion. Such adhesion relies on several molecular mechanisms and factors expressed in both the niche cells and stem cells. E-cadherin is a well-known example of such a cell adhesion molecule. In general, cadherins can modulate cell adhesion by interacting with intra- and extracellular domains, linking cells to cytoskeleton-associated proteins and nearby cells [43]. In doing so, cadherins can form strong AJs between cells. Cadherins have been found in stem cells in several types of tissues including brain stem cells and HSCs. Some cadherins can even regulate gene expression and signaling pathways by interacting with certain proteins and receptors. For example, N-cadherin can regulate the signaling by interacting with FGF receptors and also affect HSCs into quiescence by a similar mechanism.

In addition to cadherins, stem cells can also express integrins which are transmembrane molecules that modulate ECM interactions. Integrins can bind to ECM through certain proteins (e.g., collagen and laminin) and bind to surface adhesion molecules (e.g., CD54) and vascular cell adhesion molecules (e.g., CD106). Due to their ability to modulate cell adhesions, both cadherins and integrins are essential for spindle orientation of certain tissues (e.g., skin and brain) where the spindle of stem cells determines the type of division (symmetric or asymmetric division). It has been shown that the removal of such molecules can easily translate into randomized orientations. Other cellular mechanisms such as cell polarity, maintenance and signaling pathways can also be affected by adhesion molecules; therefore, it is difficult to draw a clear link between cellular adhesion and other cellular events. Other molecules that play a role in stem cells adhesion include Connexin 43 (a junction protein that support HSCs in stroma and affect CXCL12 secretion) and Vcam 1 [14].

Cancer-induced inflammation is a hallmark in solid tumor progression and considered to be essential in modifying their microenvironment. Although chemokines and cytokines, tumor surrounding cells (e.g., endothelial, mesenchymal, pericytes and fibroblast cells) can communicate directly with immune cells such as macrophages, neutrophiles, NK cells, T and B lymphocytes and other immune cells. Such interactions and the level of activity of each type of cell determine the resulting immune responses (pro or anti-tumor). For example, difference with tumor-induced inflammation is that it takes the profile of chronic inflammatory responses and uses cytokines and chemokines not only to communicate but also to recruit immune elements in order to resemble an unhealed wound. Consequently, the niche would remain in tumor favoring conditions such as hypoxia and EMT to enhance the survival and functioning of cells [9].

A complicated network of molecules is therefore responsible for the inflammatory response in the niche, and some of these are also involved in other pathways and functions. IL-6, IL-8, TGF-β, NFκ and TNF-α are some of the elements shown to play a role in the tumor-induced inflammatory environment. IL-6 is an important cytokine linked to proliferation, differentiation, and maturity of cells, making this molecule a promising target for many anti-tumor therapies. In CSCs, IL-6 has been found to play a role in Notch-3 regulation that leads to the malignant behavior of cells in human ductal breast carcinoma. The role of IL-6 in regulating CSCs was also studied in other types of human cancer (e.g., glioblastoma), where targeting it led to cellular apoptosis and reduction in tumor growth. NFκB pathway has also been found to be activated by inflammatory cytokines in the CSC niche. This pathway plays a role in modulating EMT factors Twist, Snail, and Slug. Therefore, NFκB is important for CSC migration and invasion in human cancers (e.g., pancreas, skin, and ovarian cancer) [1].

Current CSC-associated studies focus on their molecular features and differences and discovered novel markers including CD44, CD133, and CD24 [28, 29, 44]. However, markers specificity is still a challenge, since many are shared with normal stem cells and progenitors [3]. Several markers have already been linked to certain types of CSCs (breast cancer: CD44^+^/CD24^−^/ALDH^+^; leukemia: CD34^+^/CD8^−^; liver: CD90^+^; pancreas: CD44^+^/CD24^+^/ESA^+^; epidermal surface antigen) [11]. Therefore, more efforts are required to identify CSC markers as there are contradicting reports regarding the exact role of markers in CSCs [6].

### Genetics and epigenetic modulations in the CSC niche

The genetic diversity and heterogeneity of cancers has led to exploration of their genetic background and importance in CSC niches. Differences in genetic features or mutations among CSC clones can determine their phenotype (such as the level of activeness or dormancy, which can directly affect the survival of CSCs against chemotherapies) [45]. With the bidirectional relation between CSCs and their niches, genetic alterations on both sides can affect or determine the fate of CSCs or their link to the niche. This shows the advantage of the slow cycling of CSCs which helps avoid genetic mutations (which might not fit with the niche requirements) [3, 46]. So far, two models prevail in explaining the intratumor heterogeneity, namely the hierarchical model which proposes that CSCs are originally organized in their niches in a cellular hierarchy similar to normal niches and the clonal evolution model which suggests that different somatic mutations and epigenetic alterations are acquired by different sub-clones of CSCs during tumor evolution, leading to molecular and biological differences among clones [8]. CSC niche conditions also influence the DNA profile and stability; for example, severe hypoxia leads to DNA replication arrests, which introduces different biological actions within the cells and adds to the intratumor heterogeneity [47, 48]. As mentioned earlier, several transcriptional factors and signaling pathways can also affect the genetic profile of CSCs [49].

DNA methylation is a type of common epigenetic modification, and evidence is accumulating, suggesting that DNA methylation is a critical epigenetic reprogramming mechanism that may play a crucial role in CSCs biology [50]. BEX1, under the regulation of DNA methyltransferases 1 (DNMT1), was shown to have differential expression levels in Hepatoblastoma, CSC-hepatocellular carcinoma (HCC), and non-CSC HCC patients. This is essential for the self-renewal and maintenance of liver CSCs through activation of Wnt/β-catenin signaling [51]. Huang W et al. performed whole-genome bisulfite sequencing on tumor-repopulating cells (TRC), which are cancer stem cell (CSC)-like cells with highly tumorigenic and self-renewing abilities, their results showed that CSCs markers were biased toward altering their methylation in non-CG methylation and enriched in the gene body region, indicating non-CG DNA methylation plays a vital role in TRC selection [52].

Ubiquitination is also an important post-translational modification for CSCs self-renewal, maintenance, differentiation and tumorigenesis [53]. A recent study showed that targeting MYH9 could block HBX-induced GSK3β ubiquitination to activate the β-catenin destruction complex and then further suppress cancer stemness and EMT in hepatocellular carcinoma [54].

Besides, miRNAs and lncRNAs are also important modulating elements in the renewal and differentiation of CSCs. It has been shown that the loss of some miRNAs or lncRNAs can lead to the failure of the CSCs’ functioning (including the ability of CSCs to regulate stem cell markers, transcriptional factors, and binding proteins). Such changes affect the stemness and, therefore, the identity of CSCs. Interference with miRNAs or lncRNAs affects the role of cytokines and chemokines in CSCs, risks the whole balance of the CSC niche and influences the fate of those cells [11, 55–58].

### Signaling pathways regulating the CSC niche

**Fig. 4:**
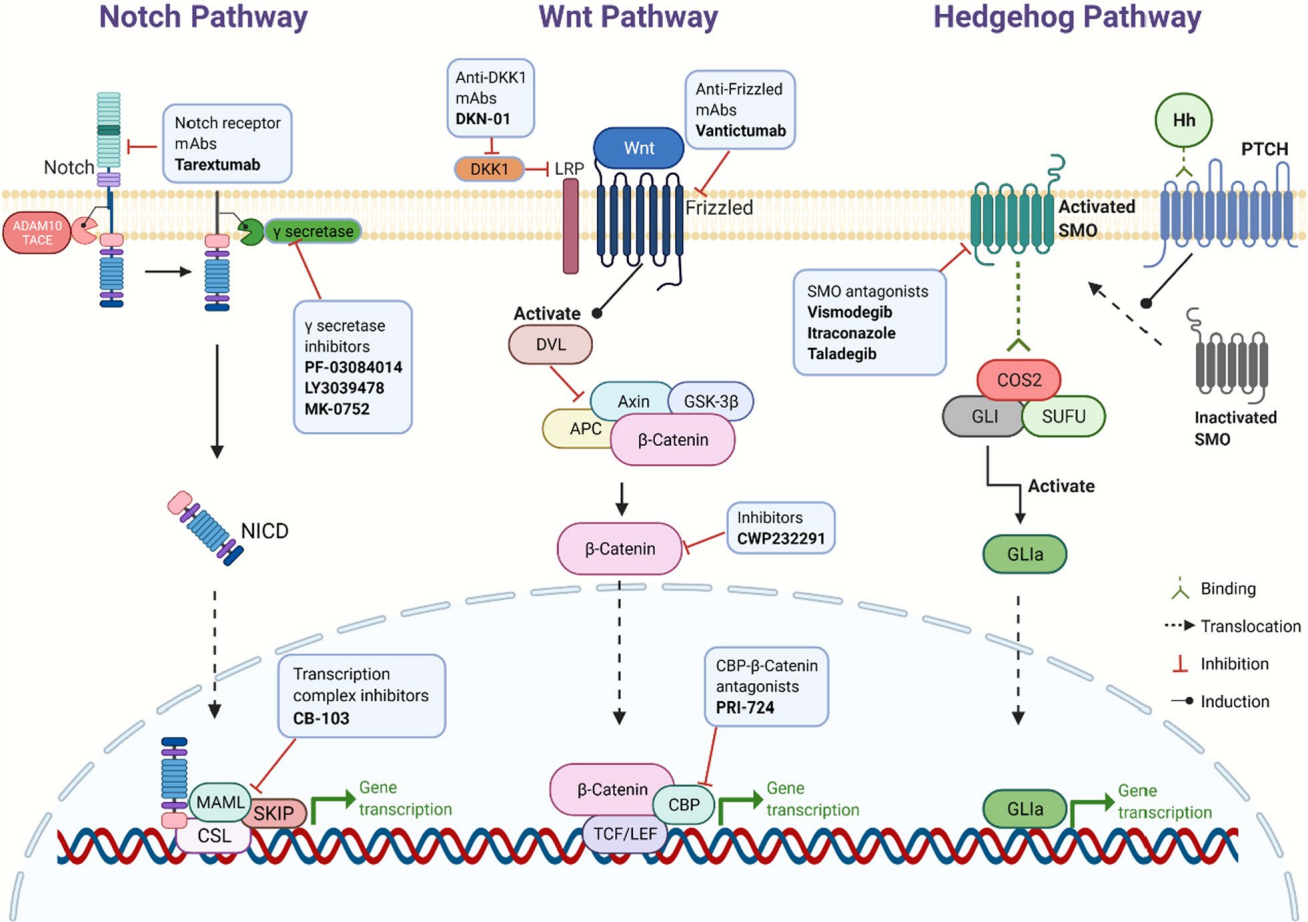
Key signaling pathways and related targeted inhibitors in the CSC niche. Notch pathway: After twice cleavages by ADAM10/TACE and γ-secretase, respectively, Notch intracellular domain (NICD) is finally released. NICD could translocate into the nucleus and interact with the transcription complex, promoting Notch target gene expression. Wnt pathway: When Wnt proteins bind to the Frizzled family receptors, dishevelled protein (DVL) is activated. DVL would inactivate the multiprotein destruction complex (including APC, Axin, and GSK-3β), which could lead β-catenin to degradation. With the DVL activation, β-catenin starts to accumulate in the cytoplasm. Part of β-catenin enters the nucleus to interact with TCF/LEF and CBP, promoting the expression of target genes. Hedgehog pathway: Binding PTCH with Hh would translocate smoothened (SMO) to the cell membrane and activate it. The activated SMO could easily bind to the COS2/GLI/SUFU complex, then GLI releases from the complex, referred to as GLIa. GLIa activates the transcription of downstream target genes

The CSC niche contains many components that could maintain CSCs in a quiescence state and regulate the cell plasticity and quiescence by induction of several signaling pathways. Such signaling pathways in the CSC niche are the map or the network of all the interactions among cells and components in the niche. The importance of signaling pathways covers most characteristics of CSCs (e.g., self-renewal, proliferation, differentiation, metastasis, cell cycling, angiogenesis, and tumorigenesis) [15, 59]. It has already been shown that several pathways in various types of niches such as Wnt, TGFβ, BMP, JAK-STAT, PI3K, and cell cycle pathways [60]. Signaling pathways in the CSC niche depend on the activation of other niche components (such as growth factors). In addition, many of these pathways act in the short term, thus requiring the CSCs to stay in the niche in order to benefit from the signaling [14]. Here, we highlight the common pathways often observed in the CSC niche (Fig. 4).

The Notch pathway is a well-known example in the interactions within the CSC niche. In this pathway, Notch binds to certain ligands that eventually translocate to the nucleus to activate cycling inhibitors (e.g., p21) [9]. Although the Notch pathway is responsible for the development of normal cells and maintenance of cellular proliferation and apoptosis, it is altered in several cancers (e.g., HCC) and can activate tumor initiation and progression. It has been shown that the Notch pathway is regulated by several genes including the tumor suppressor gene RUNX3, which explains the relation between Notch and tumor growth [59]. The Notch pathway is involved in the adhesion of CSCs in the niche, making it difficult to draw a clear line between the role of Notch in signaling and adhesion, especially in niches such as those in skin tissues [61, 62]. All these functions of the Notch pathway make it a promising target of anti-tumor therapeutic approaches, regardless of its non-specific CSC nature. For example, blocking Notch-4 in breast cancer affected the ability of new cells formation [15].

The Wnt is another pathway and is similar to Notch as it is involved in the development and a functioning regulation of normal stem cells and therefore strongly correlates with CSCs. The hyper activation of Wnt has been shown to initiate cancer in the pituitary gland in animal models and increases the CSCs’ symmetrical division [63–65]. In HCC in particular, the Wnt/β-catenin pathway activation increases CSC activity and cells proliferation. Such an activation is controlled by PTEN, which is a tumor suppressor gene. Studies have shown that controlling or inhibiting the Wnt/β-catenin pathway can elevate the efficiency of drugs and other therapeutic approaches [66–68]. This pathway is also a potential target for future molecular therapies, since it depends on the concentration of β-catenin within the cytoplasm [69]. Together with Notch, Wnt is also involved in stemness stimulation in CSCs, which is essential for the identity of these cells [37, 70].

The Hedgehog (Hh) pathway also plays a pivotal role in the CSC niche. In addition to its involvement in the development of cancer, it plays a role in determining the invasiveness and histological differentiation in tumors. Studies found that the simultaneous activation of the Hh pathway and EMT may be a reason for the chemoresistance that HCC cells usually exhibit [59]. Many drugs have been found to target Hh pathway (e.g., vismodegib, itraconazole, cyclopamine, and PTCN antibodies) to regulate CSC functions and inhibit tumor growth [11, 15].

Another CSC controlling pathway is TGF-β. A recent study showed that tumor-initiating cells play a crucial role in creating a CSC niche microenvironment, which is required for cancer progression and therapy resistance [37]. Tumor cells release the interleukin-33 cytokine, which promotes the differentiation of the myeloid cells into macrophages. In turn, these macrophages further send TGF-β signals to CSCs, promoting their malignant progression and therapy resistance [71]. Other important pathways include TF pathway [72], Hippo pathway [73], and cell cycle pathways [74].

## The CSC niche in different cancers

CSC niches have already been identified in several types of human cancers including brain, liver, breast, lung, head and neck, prostate, melanoma, gastric, pancreatic, renal, ovarian, esophageal and colorectal cancers [75–87]. Various studies have focused on the similarities and differences among the niches in different cancers and how cancer specific microenvironments can affect the formation and the development of CSC niches [4, 6, 88–90]. Here, we review some of the important examples of CSC niches in major cancers in order to further understand the interaction between tumors and CSC niches, and how the previously mentioned molecules and mechanisms participate in different tumors.

In general, the complexity of hepatic cancers posed a challenge for researchers since different tissues showed different sets of CSC markers and expression patterns [91, 92]. To initiate a tumor in the liver, a CSC needs to survive the journey to the liver through the hepatic microvesiculation where it faces several intravascular death risks. CSCs therefore have to inhibit the liver immune responses to successfully complete clinical metastasis. In order to succeed, CSCs require several mechanisms and changes in the liver microenvironment, such as the inhibition of proteases by TIMP-1 which enables the formation of pre-metastatic niches and the expansion of metastasis. Hypoxia and HIF-1 can also activate HGF and its receptor Met which play a role in the development of liver metastasis. It has also been found that Kupffer cells and neutrophils in the niche increase the production of TNF-α, IL-1, and MMP-9 [68]. EGFR, VEGF, PDGF, TNF, SDF1, and other angiogenic factors enhance the growth and survival of hepatic cancer stem cells (HCSCs) and their resistance to radio- and chemotherapies [93–96].

So far, several HCSCs markers have been proposed (e.g., CD133, CD90, CD24, CD13, EpCAM, ALDH, and OV6) and current research efforts aim to link different combinations of these markers in HCSCs to the differences in phenotypes among CSC populations. For example, CD90^+^ cells have been found to upregulate genes related to inflammation and drug resistance while CD133^+^ cells have Akt/PKB pathway activation which translate into suppression of apoptosis and resistance to radiotherapy [59].

Studies showed that post surgery, any remaining HCSCs may be re-activated and even invade the vascular system leading to recurrence and metastasis of the primary tumor [97]. As for chemotherapy, HCSCs are more resistant to anti-tumor drugs including doxorubicin and 5-flouracil (5-FU). Such drugs can also alter the expression of certain genes (e.g., ABCG2) which increases the proliferation rate of HCSCs. Many agents are being developed to target certain molecules and markers of HCSCs, including AKT1 inhibitors, CD44 neutralizing antibodies, exogenous BMP4, pimozide, cylopamine, and other agents. Markers including CD133 and EpCAM also show great potential in the creation of specific targeted therapies as well as novel strategies targeting the crosstalk between the stroma and the tumor [59].

Gastric cancer is another type of malignancy with increasing incidence and mortality. Gastric cancer stem cells (GCSCs) have already been identified in different cell lines with markers including CD44, CD54, CD24, and CD71. Interestingly, many reports indicate the interaction between Helicobacter Pylori (HP) infection and GCSCs, such infection leads to modulation of stem cells into GCSCs in animal models [88, 98]. The chronic inflammation caused by such an infection is also helpful for the GCSC niche. Since treatments such as surgery, chemotherapy, radiation, and immunotherapy are often challenged with the problem of tumor relapse, GCSCs have been proposed as potential targets for drugs that inhibit their stemness and functions. For example, Trastuzumab targets CD90^+^ GCSC and decreases the growth of gastric cancer tissues (when used with chemotherapy) [99]; Salinomycin targets ALDH^+^ GCSC (a cell population which is resistant to chemotherapy drugs such as 5-FU and CDDP) [100].

In the case of breast cancer, it has been found that different populations of breast CSCs (BCSCs) can generate different lines of tumor cells. Yet, the genotype of the new cells may not completely resemble the original CSC genetic profile, which may indicate a number of mutations along the way [8]. It has been shown that BRCA1 expression (well-known tumor suppressor gene in breast cancer) is decreased by transcription factors such as Slug, while at the same time Slug stability is reduced by BRCA1 gene expression. This negative feedback-based balance is a key element in maintaining a normal growth of tumor and determining the level of stemness of niche cells [101, 102]. Pluripotency factors including SOX are also over expressed in BCSC niches and other than their role in EMT and metastasis, the SOX family upregulates the expression of EZH2 which is important for histone methylation for several genes and can activate Raf1-β-catenin pathway [103, 104]. With the identification of drugs that can specifically target BCSCs, essential pathways including Wnt can be inhibited. BCSCs resistance to radiotherapy can also be solved by advanced approaches including gold Nano-shell-mediated hyperthermia therapy [105].

In hematopoietic malignancies, HCSCs (also known as leukemia stem cells, LSCs) have been identified in acute myeloid leukemia (AML) in the 1990s, with specific markers such as CD34^+^/CD38^−^ [45, 106]. Although the interaction and communication mechanism between LSCs and their niche is not as clear as in solid tumors, targeting the niche may still be useful as it can affect the mobility of LSCs before escaping the original niche. This theory is supported by the expression of certain chemokines and receptors in the niche such as CXCR4 and CXCL12 [107]. In order to prevent relapse of primary cancer, targeting the niche might be of major therapeutical importance, as it provides a sanctuary for LSCs against conventional therapies [108].

The chemotherapeutic drugs cytarabine and daunorubicin have been the standard choices for many years, despite the risks of relapse or therapeutic resistance. As a result, there is an urgent need for novel therapies addressing the potential role of LSCs. In animal models, G-CSF treatment has shown promising results as it stimulates dormant LSCs and activated their cell cycle, leading to increased sensitivity to chemotherapy. Such treatments still require more development, but they certainly create new hope in this field [107].

In other examples of cancer, malignant bone tumors usually include osteosarcoma, multiple myeloma, and solid tumor metastasis. Bone metastasis pose a significant challenge as it is in general incurable and is associated with resistance to chemotherapy. Other than migrated tumor cells, the bones are also the source of HSCs. It has been shown that these two different populations of cells compete for the same niches in the bones, suggesting that stem cell niches may hold a great potential in the future as a target of treating metastatic tumors [109]. Although tumor stem cells can alter the microenvironment of the bone and the existing hematopoietic niches, the niche can still distinguish a normal stem cell from a CSC. Consequently, modulating the niche in favor of normal SC compared to CSC can prevent the invasion of migrated cells [110].

## Therapeutic strategies targeting the CSC niche

Therapy resistance and tumor relapse are two major obstacles in the clinical management of solid cancers [111]. There is evidence that many residual tumors are enriched with CSCs after therapy [69, 112]. Thus far, this phenomenon has been explained by the theory that even after a radical resection of tumor lesions (*R*0) by surgery and applying chemotherapy and radiotherapy, some CSCs still manage to escape and survive due to their therapy resistance [11, 113]. CSCs are usually quiescent and slow cycling and have an active anti-apoptotic machinery [114], efficient DNA repairing systems (DNA checkpoint kinases) and stabilized stemness features, all of which may contribute to their resistance [115]. To date, many chemoresistance mechanisms have been found across distinct cancer types such as hypoxia, EMT and the signaling pathways which regulate CSC function, for instance Notch, Wnt and Hedgehog signaling. The recent clinical trials targeting CSCs or CSC-associated pathways are shown in Table 1.

**Table 1:** The recent clinical trials targeting CSCs or CSC-associated pathways

Pathway	Agent	Tumor type	Phase	Study number
EMT	Plerixafor	Pancreatic cancer	Phase 2	NCT04177810
		Multiple myeloma	Phase 4	NCT05087212
	Eribulin	Breast cancer	Phase 1	NCT02120469
			Phase 2	NCT04517292
	GI-6301	Chordoma	Phase 2	NCT02383498
	Vorinostat	T-cell lymphoma	Phase 3	NCT01728805
	Fresolimumab	Breast cancer	Phase 2	NCT01401062
Hypoxia	Temsirolimus	Renal cell carcinoma	Phase 1	NCT00700258
	CRLX101	Prostate cancer	Phase 2	NCT03531827
		Renal cell carcinoma	Phase 2	NCT02187302
Notch	PF-03084014	Desmoid tumors	Phase 2	NCT04195399
		Breast cancer	Phase 1	NCT01876251
	LY3039478	Solid tumors	Phase 1	NCT02836600
	MK-0752	Breast cancer	Phase 4	NCT00756717
	Tarextumab	Solid tumors	Phase 1	NCT01277146
	CB-103	Advanced cancers	Phase 1/2	NCT03422679
Wnt	PRI-724	PDAC	Phase 1	NCT01764477
	DKN-01	Biliary tract cancer	Phase 1	NCT04057365
		Gastric cancer	Phase 2	NCT04363801
		Liver cancer	Phase 1/2	NCT03645980
	Vantictumab	Breast cancer	Phase 1b	NCT01973309
	CWP232291	Acute myeloid leukemia	Phase 1/2	NCT03055286
Hedgehog	Vismodegib	Basal cell carcinoma	Phase 2	NCT02667574
		Chondrosarcomas	Phase 2	NCT01267955
		Meningiomas	Phase 2	NCT02523014
		Medulloblastoma	Phase 2	NCT01878617
	Itraconazole	Esophageal cancer	Phase 2	NCT04481100/NCT04018872
		Prostate cancer	Phase 1/2	NCT03513211
	Taladegib	Solid tumors	Phase 2	NCT05199584

As described before, EMT is closely associated with the biological features of the CSC, including stemness, immune escape and resistance to chemotherapy [116]. Recent studies have shown that EMT plays a major role in therapy resistance [117–119]. Diverse factors and cytokines are involved in regulation of EMT, complicating targeted therapy approaches. For instance, responses to chemotherapy in a large group of breast cancer patients revealed a close association between chemoresistance and increased stromal gene expression, and this transcriptional upregulation appeared to be caused by activation of the EMT, which suggested anti-stromal agents may offer a new strategy to overcome therapy resistance [120]. A Phase I/II clinic trial showed that Plerixafor, as an anti-stromal agent which is an important inhibitor of CRCX4, combined with bortezomib restored the chemosensitivity in relapsed multiple myeloma, which suggested that the novel therapy may target the tumor microenvironment and overcome resistance to therapy [121]. In addition, Kari et al. established an EMT lineage-tracking system in breast-to-lung metastasis mice models, using a mesenchymal-specific Cre-mediated fluorescent marker switch system. They found out that EMT cells significantly contribute to recurrent lung metastasis formation after chemotherapy [122]. According to these capacities of the EMT, creating more effective treatment strategies to directly target the EMT program is promising and urgently needed.

Moreover, TGFβ signaling is one of the characterized pathways involved in EMT induction, as enhanced TGFβ signaling in cancer accelerates EMT program and maintains a highly proliferative phenotype [123]. Accumulating research studies have demonstrated that TGFβ-mediated EMT may initiate and facilitate therapy resistance, which is inhibited by blockade of TGFβ [124–126]. Fresolimumab, a TGFβ blocking antibody, showed an effective clinical application which was demonstrated in many clinical trials [127–129]. Furthermore, two doses of fresolimumab were explored in metastatic breast cancer in a prospective randomized trial, patients receiving the higher fresolimumab dose had a better systemic immune response and longer median overall survival compared to the lower dose group [130]. Therefore, TGFβ blockade might be a feasible and promising strategy for a CSC targeted therapy.

Additionally, another potential therapeutic target for preventing the induction of EMT is hepatocyte growth factor (HGF)-HGR receptor (HGFR, MET) signaling, which contributes to cancer pathogenesis, as exemplified by its frequent activation, often by point mutation or amplification of the MET, in many cancer types [131]. Therefore, targeting HGR or HGR-MET signaling is a promising therapeutic method which may also regulate CSCs. Recently, many studies show that anti-MET antibodies prevent HGF binding to MET and, subsequently, inhibit cancer progression and restore the chemoresistance, as well as improve the therapeutic efficiency [132–134].

In addition, hypoxia contributes to therapy resistance via a number of mechanisms, including maintaining CSC stemness, function of HIFs, drug transporters and affecting EMT [135]. Targeting these key factors is considered as a promising strategy for anti-CSC therapy. Acriflavine, a potent inhibitor of HIF-1α dimerization, could disturb glucose metabolism in melanoma regardless of the hypoxic condition [136]. Chetomin, another inhibitor of HIF-1α, could suppress the transcriptional activity via targeting p300 recruitment [137]. Furthermore, other agents, such as wortmannin, temsirolimus and camptothecin, have been proved to inhibit HIF-1α protein translation by targeting corresponding genes [138]. In addition, hypoxia can also mediate chemoresistance of CSCs by drug efflux through drug transporters [ATP-binding cassette (ABC) family membrane transporters]. ABCB1 and ABCG2 (BCRP), as the well-known ABC family transporters, have been reported to be induced in hypoxia [139, 140]. A recent study showed that vatalanib, a multitargeted tyrosine kinase inhibitor for all isoforms of VEGFR, PDGFR and c-Kit, makes ABCB1 and ABCG2-overexpressing multidrug-resistant colon cancer cells sensitive to chemotherapy in hypoxic microenvironment. Utilizing the combination of vatalanib with conventional anti-tumor drugs might be a promising tool to restore the chemosensitivity in colon cancer treatment [141]. Similar mechanism is found in ovarian cancer stem cells: Hypoxia-induced HIF-2α overexpression endows ovarian cancer stem cells with resistance to adriamycin by promoting BCRP expression and adriamycin efflux [139].

We have described the Notch, Wnt and Hedgehog signaling pathways in CSC regulations before, which are widely explored in current CSC target therapies (Fig. 4). γ-secretase inhibitor (GSI), as a major clinical method used to inhibit Notch signaling, has been proved in many clinical trials. Combining trastuzumab plus a GSI has been reported to reduce recurrence rate for ErbB-2-positive breast tumors [142]. Another clinical trial shows that pharmacologic inhibition of the Notch pathway can reduce CSCs in breast cancer models [143]. A phase I study shows that ipafricept (OMP-54F28, a Decoy Receptor for Wnt Ligands) can be safely administered with manageable toxicities [144]. In addition, HH ligands, SMO and GLI transcription factors are three main targets of Hh pathway antagonists [60], whose effectiveness in basal cell carcinoma and prostate cancer was already shown in many trials [145–147].

In the past few years, CSC-directed immunotherapy has emerged. Besides the antibodies targeting CSC-related signaling pathways mentioned above, novel anti-CSC immunotherapeutic approaches, such as cancer vaccines, checkpoint inhibitors and chimeric antigen receptor T-cell (CAR-T cell) therapies, have been developed [148, 149]. One personalized neoantigen dendritic cell vaccine was chosen to treat metastatic lung cancer (NCT02956551). In this trial, the disease control rate was 75% and the median overall survival was 7.9 months; the combination therapy of immune checkpoint inhibitors and the vaccines had a better progression free survival (2.2 vs 12.2 months) and overall survival (7.6 vs 11.2 months) trend; this study provided new evidence for cancer vaccine therapy and promising therapeutic opportunities for lung cancer treatment [150]. In addition, as a novel therapeutic strategy, CAR-T cell therapy is widely used in many types of cancer, especially against hematologic malignancies. CD19/CD22/CD33, been detected in leukemia patients, is a noteworthy target for immune-cellular therapy against AML [151–154]. The ongoing clinical trials of CAR-T cell therapy are listed in Table 2.

**Table 2:** CAR-T cell therapy in ongoing clinical trials

Target	Tumor type	Phase	Study number
CD19	Relapsed/refractory B-ALL	Phase 2	NCT05334823
	Relapsed/refractory leukemia/lymphoma	Phase 1	NCT03984968
CD19/CD22	Non-Hodgkin lymphoma	Phase 1	NCT05098613
CD30	Relapsed/refractory Hodgkin lymphoma	Phase 2	NCT04268706
CD33	Acute myeloid leukemia	Phase 1/2	NCT03971799
CD38	Acute myeloid leukemia	Phase 1	NCT05239689
CD70	Pancreatic/renal/breast cancer	Phase 1/2	NCT02830724
CD123	Acute myeloid leukemia	Phase 1	NCT04230265
CD171	Neuroblastoma	Phase 1	NCT02311621
c-Met/PD-L1	Hepatocellular carcinoma	Phase 1	NCT03672305
EpCAM	Pancreatic/gastric/colorectal cancer	Phase 1	NCT05028933
GD2	Neuroblastoma	Phase 1	NCT01822652
GPC3	Liver cancer	Phase 1	NCT02932956
	Hepatocellular carcinoma	Phase 1	NCT02905188
MOv19-BBz	Ovarian/fallopian tube/peritoneal cancer	Phase 1	NCT03585764
P-MUC1C-ALLO1	Advanced or metastatic solid tumors	Phase 1	NCT05239143
IL13Rα2	Brain tumor	Phase 1	NCT04661384

The current studies have confirmed that CSCs are considered as the root of cancer relapse, metastasis and therapy resistance, while further research is required for the development of more effective strategies to eradicate CSCs.

## Conclusion

Recent studies have expanded our knowledge regarding CSCs and their niche. The novel markers and mechanisms in CSC niches as greatly improved our understanding of cancer and its microenvironment. It also changed the proposition that metastasis is a late-stage complication of cancer and explains the long-standing dilemma of why a tumor may relapse even after a complete surgical removal, chemotherapy or radiotherapy. Regardless to the improvements in this field, we still need more efforts to annotate CSC niches regarding molecular interactions and mechanisms controlling their stemness capacity and relationship with dormancy and chemo-immuno resistance. More challenges are ahead before we can move forward from basic to translational research and further clinical applications; nonetheless, such findings may bring a great benefit for millions of patients and guide future anti-cancer therapeutic strategies.

## Data Availability

Not applicable.

## Abbreviations

ABCG2: ATP-binding cassette sub-family G member 2
ADAM10: A disintegrin and metalloproteinase domain-containing protein 10
AJs: Adherens junctions
AKT1: RAC-alpha serine/threonine-protein kinase
ALDH: Aldehyde dehydrogenate
AML: Acute myeloid leukemia
APC: Adenomatous polyposis coli
ATP: Adenosine triphosphate
Axis: Axis inhibition protein
B-ALL: B-cell acute lymphoblastic leukemia
BCSCs: Breast cancer stem cells
bHLH–PAS: Basic helix loop helix Per Arnt Sim
BMP: Bone morphogenetic proteins
BRCA1: BReast-CAncer susceptibility gene 1
CAFs: Cancer-associated fibroblasts
CBP: CREB-binding protein
CD: Cluster of differentiation
CSCs: Cancer stem cells
CSF-1: Colony stimulating factor 1
CXCL12: C-X-C motif chemokine 12 also known as SDF1
CXCR4: C-X-C chemokine receptor type 4
DVL: Dishevelled protein
ECM: Extracellular matrix
EGF: Epidermal growth factor
EMT: Epithelial to mesenchymal transition
EpCAM: Epithelial cell adhesion molecule
ER: Endoplasmic reticulum
ESA: Epidermal surface antigen
EZH2: Enhancer of zeste homolog 2
FGF: Fibroblast growth factor
5-FU: 5-Flouracil
GCSCs: Gastric cancer stem cells
GLI: Glioma-associated oncogene
GLUT-1: Glucose transporter 1
GSK-3β: Glycogen synthase kinase 3 beta
HCC: Hepatocellular carcinoma
HCSCs: Hepatic cancer stem cells
HGF: Hepatocyte growth factor
Hh: Hedgehog pathway
HIF: Hypoxia inducible transcription factor
HP: Helicobacter pylori
HSCs: Hematopoietic stem cells
IGF-1: Insulin-like growth factor-1
IL-6: Interleukin-6
JAK: Janus kinases
LSCs: Leukemia stem cells
MDSC: Myeloid-derived suppressor cell
MET: Mesenchymal epithelial transition
HGFR: Hepatocyte growth factor receptor
miRNA: Micro-RNA
MMP-3: Matrix metalloproteinase 3
MSC: Mesenchymal stem cell
mTOR: Mammalian target of rapamycin
NFkB: Nuclear factor Kappa B
NICD: Notch intracellular domain
NK: Natural killer cell
Oct4: Octamer-binding transcription factor 4
PDGF: Platelet derived growth factor
PI3K: Phosphoinositide 3-kinase
PTCH: Protein patched homolog
PSF1: Partner of SLD five 1
PTEN: Phosphatase and tensin homolog
ROS: Reactive oxygen species
RUNX3: Runt-related transcription factor 3
SALL4: Sal-like protein 4
SDF-1: Stromal cell-derived factor-1
Six1: Sineoculis homeobox homolog 1
SMO: Smoothened
Snail: Zinc finger protein SNAI1
STAT: Signal transducer and activator of transcription proteins
TACE: Tumor necrosis factor-α converting enzyme
TAM: Tumor-associated macrophages
TAN: Tumor-associated neutrophils
TCF/LEF: T cell factor/lymphoid enhancer factor
TF: Tissue factor
TGF-β: Transforming growth factor-beta
TIMP-1: Tissue inhibitor of metalloproteinases 1
TNF-α: Tumor necrosis factor alpha
TPA: Tissue polypeptide antigen
Vcam 1: Vascular cell adhesion molecule 1
VEGF: Vascular endothelial growth factor
Zeb: Zinc finger E-box binding homeobox

## References

[1] Cabarcas SM, Mathews LA, Farrar WL (2011). The cancer stem cell niche–there goes the neighborhood?. Int J Cancer.

[2] Kordes C, Häussinger D (2013). Hepatic stem cell niches. J Clin Invest.

[3] Sottocornola R, Lo CC (2012). Dormancy in the stem cell niche. Stem Cell Res Ther.

[4] Lacina L, Plzak J, Kodet O, Szabo P, Chovanec M, Dvorankova B, Smetana K (2015). Cancer microenvironment: what can we learn from the stem cell niche. Int J Mol Sci.

[5] Stine RR, Matunis EL (2013). Stem cell competition: finding balance in the niche. Trends Cell Biol.

[6] Ribatti D (2012). Cancer stem cells and tumor angiogenesis. Cancer Lett.

[7] Takakura N (2012). Formation and regulation of the cancer stem cell niche. Cancer Sci.

[8] Guo W (2014). Concise review: breast cancer stem cells: regulatory networks, stem cell niches, and disease relevance. Stem Cells Transl Med.

[9] Shigdar S, Li Y, Bhattacharya S, O'Connor M, Pu C, Lin J, Wang T, Xiang D, Kong L, Wei MQ (2014). Inflammation and cancer stem cells. Cancer Lett.

[10] Singh SR (2013). Cancer stem cells: recent developments and future prospects. Cancer Lett.

[11] Yu Z, Pestell TG, Lisanti MP, Pestell RG (2012). Cancer stem cells. Int J Biochem Cell Biol.

[12] Plaks V, Kong N, Werb Z (2015). The cancer stem cell niche: how essential is the niche in regulating stemness of tumor cells?. Cell Stem Cell.

[13] Batlle E, Clevers H (2017). Cancer stem cells revisited. Nat Med.

[14] Chen S, Lewallen M, Xie T (2013). Adhesion in the stem cell niche: biological roles and regulation. Development.

[15] Yi SY, Hao YB, Nan KJ, Fan TL (2013). Cancer stem cells niche: a target for novel cancer therapeutics. Cancer Treat Rev.

[16] Mohyeldin A, Garzón-Muvdi T, Quiñones-Hinojosa A (2010). Oxygen in stem cell biology: a critical component of the stem cell niche. Cell Stem Cell.

[17] Liu L, Michowski W, Kolodziejczyk A, Sicinski P (2019). The cell cycle in stem cell proliferation, pluripotency and differentiation. Nat Cell Biol.

[18] Watt FM, Huck WT (2013). Role of the extracellular matrix in regulating stem cell fate. Nat Rev Mol Cell Biol.

[19] Janiszewska M, Primi MC, Izard T (2020). Cell adhesion in cancer: beyond the migration of single cells. J Biol Chem.

[20] Peitzsch C, Perrin R, Hill RP, Dubrovska A, Kurth I (2014). Hypoxia as a biomarker for radioresistant cancer stem cells. Int J Radiat Biol.

[21] Jing X, Yang F, Shao C, Wei K, Xie M, Shen H, Shu Y (2019). Role of hypoxia in cancer therapy by regulating the tumor microenvironment. Mol Cancer.

[22] Alsaab HO, Sau S, Alzhrani RM, Cheriyan VT, Polin LA, Vaishampayan U, Rishi AK, Iyer AK (2018). Tumor hypoxia directed multimodal nanotherapy for overcoming drug resistance in renal cell carcinoma and reprogramming macrophages. Biomaterials.

[23] Rovida E, Peppicelli S, Bono S, Bianchini F, Tusa I, Cheloni G, Marzi I, Cipolleschi MG, Calorini L, Sbarba PD (2014). The metabolically-modulated stem cell niche: a dynamic scenario regulating cancer cell phenotype and resistance to therapy. Cell Cycle.

[24] Choudhry H, Harris AL (2018). Advances in hypoxia-inducible factor biology. Cell Metab.

[25] Ai Z, Lu Y, Qiu S, Fan Z (2016). Overcoming cisplatin resistance of ovarian cancer cells by targeting HIF-1-regulated cancer metabolism. Cancer Lett.

[26] Haase VH (2013). Regulation of erythropoiesis by hypoxia-inducible factors. Blood Rev.

[27] Schito L, Semenza GL (2016). Hypoxia-inducible factors: master regulators of cancer progression. Trends Cancer.

[28] Won C, Kim BH, Yi EH, Choi KJ, Kim EK, Jeong JM, Lee JH, Jang JJ, Yoon JH, Jeong WI (2015). Signal transducer and activator of transcription 3-mediated CD133 up-regulation contributes to promotion of hepatocellular carcinoma. Hepatology.

[29] Bai J, Chen WB, Zhang XY, Kang XN, Jin LJ, Zhang H, Wang ZY (2020). HIF-2α regulates CD44 to promote cancer stem cell activation in triple-negative breast cancer via PI3K/AKT/mTOR signaling. World J Stem Cells.

[30] Lah TT, Novak M, Breznik B (2020). Brain malignancies: glioblastoma and brain metastases. Semin Cancer Biol.

[31] de Sousa e Melo F, Kurtova AV, Harnoss JM, Kljavin N, Hoeck JD, Hung J, Anderson JE, Storm EE, Modrusan Z, Koeppen H (2017). A distinct role for Lgr5(+) stem cells in primary and metastatic colon cancer. Nature.

[32] Carvalho R, Paredes J, Ribeiro AS (2020). Impact of breast cancer cells’ secretome on the brain metastatic niche remodeling. Semin Cancer Biol.

[33] Marzagalli M, Raimondi M, Fontana F, Montagnani Marelli M, Moretti RM, Limonta P (2019). Cellular and molecular biology of cancer stem cells in melanoma: possible therapeutic implications. Semin Cancer Biol.

[34] Lytle NK, Ferguson LP, Rajbhandari N, Gilroy K, Fox RG, Deshpande A, Schürch CM, Hamilton M, Robertson N, Lin W (2019). A Multiscale map of the stem cell state in pancreatic adenocarcinoma. Cell.

[35] Oskarsson T, Batlle E, Massagué J (2014). Metastatic stem cells: sources, niches, and vital pathways. Cell Stem Cell.

[36] Barbato L, Bocchetti M, Di Biase A, Regad T (2019). Cancer stem cells and targeting strategies. Cells..

[37] Oshimori N (2020). Cancer stem cells and their niche in the progression of squamous cell carcinoma. Cancer Sci.

[38] Malanchi I, Santamaria-Martínez A, Susanto E, Peng H, Lehr HA, Delaloye JF, Huelsken J (2011). Interactions between cancer stem cells and their niche govern metastatic colonization. Nature.

[39] Mani SA, Guo W, Liao MJ, Eaton EN, Ayyanan A, Zhou AY, Brooks M, Reinhard F, Zhang CC, Shipitsin M (2008). The epithelial-mesenchymal transition generates cells with properties of stem cells. Cell.

[40] Zhang Z, Han H, Rong Y, Zhu K, Zhu Z, Tang Z, Xiong C, Tao J (2018). Hypoxia potentiates gemcitabine-induced stemness in pancreatic cancer cells through AKT/Notch1 signaling. J Exp Clin Cancer Res.

[41] Jiang N, Zou C, Zhu Y, Luo Y, Chen L, Lei Y, Tang K, Sun Y, Zhang W, Li S (2020). HIF-1ɑ-regulated miR-1275 maintains stem cell-like phenotypes and promotes the progression of LUAD by simultaneously activating Wnt/β-catenin and Notch signaling. Theranostics.

[42] Wang Z, Li Y, Sarkar FH (2010). Signaling mechanism(s) of reactive oxygen species in epithelial–mesenchymal transition reminiscent of cancer stem cells in tumor progression. Curr Stem Cell Res Ther.

[43] Kaszak I, Witkowska-Piłaszewicz O, Niewiadomska Z, Dworecka-Kaszak B, Ngosa Toka F, Jurka P (2020). Role of cadherins in cancer-a review. Int J Mol Sci..

[44] Ooki A, VandenBussche CJ, Kates M, Hahn NM, Matoso A, McConkey DJ, Bivalacqua TJ, Hoque MO (2018). CD24 regulates cancer stem cell (CSC)-like traits and a panel of CSC-related molecules serves as a non-invasive urinary biomarker for the detection of bladder cancer. Br J Cancer.

[45] Minami Y (2015). Overview: cancer stem cell and tumor environment. Oncology.

[46] Scadden DT (2014). Nice neighborhood: emerging concepts of the stem cell niche. Cell.

[47] Bristow RG, Hill RP (2008). Hypoxia and metabolism. Hypoxia, DNA repair and genetic instability. Nat Rev Cancer..

[48] Pires IM, Bencokova Z, Milani M, Folkes LK, Li JL, Stratford MR, Harris AL, Hammond EM (2010). Effects of acute versus chronic hypoxia on DNA damage responses and genomic instability. Cancer Res.

[49] Ruiz-Vela A, Aguilar-Gallardo C, Simón C (2009). Building a framework for embryonic microenvironments and cancer stem cells. Stem Cell Rev Rep.

[50] French R, Pauklin S (2021). Epigenetic regulation of cancer stem cell formation and maintenance. Int J Cancer.

[51] Wang Q, Liang N, Yang T, Li Y, Li J, Huang Q, Wu C, Sun L, Zhou X, Cheng X (2021). DNMT1-mediated methylation of BEX1 regulates stemness and tumorigenicity in liver cancer. J Hepatol.

[52] Huang W, Hu H, Zhang Q, Wang N, Yang X, Guo AY (2020). Genome-wide DNA Methylation enhances stemness in the mechanical selection of tumor-repopulating cells. Front Bioeng Biotechnol.

[53] Deng L, Meng T, Chen L, Wei W, Wang P (2020). The role of ubiquitination in tumorigenesis and targeted drug discovery. Signal Transduct Target Ther.

[54] Lin X, Li AM, Li YH, Luo RC, Zou YJ, Liu YY, Liu C, Xie YY, Zuo S, Liu Z (2020). Silencing MYH9 blocks HBx-induced GSK3β ubiquitination and degradation to inhibit tumor stemness in hepatocellular carcinoma. Signal Transduct Target Ther.

[55] Yang Q, Zhao S, Shi Z, Cao L, Liu J, Pan T, Zhou D, Zhang J (2021). Chemotherapy-elicited exosomal miR-378a-3p and miR-378d promote breast cancer stemness and chemoresistance via the activation of EZH2/STAT3 signaling. J Exp Clin Cancer Res.

[56] McCabe EM, Rasmussen TP (2021). lncRNA involvement in cancer stem cell function and epithelial-mesenchymal transitions. Semin Cancer Biol.

[57] He Y, Jiang X, Duan L, Xiong Q, Yuan Y, Liu P, Jiang L, Shen Q, Zhao S, Yang C (2021). LncRNA PKMYT1AR promotes cancer stem cell maintenance in non-small cell lung cancer via activating Wnt signaling pathway. Mol Cancer.

[58] Ni H, Qin H, Sun C, Liu Y, Ruan G, Guo Q, Xi T, Xing Y, Zheng L (2021). MiR-375 reduces the stemness of gastric cancer cells through triggering ferroptosis. Stem Cell Res Ther.

[59] Sukowati CH, Tiribelli C (2013). The biological implication of cancer stem cells in hepatocellular carcinoma: a possible target for future therapy. Expert Rev Gastroenterol Hepatol.

[60] Clara JA, Monge C, Yang Y, Takebe N (2020). Targeting signalling pathways and the immune microenvironment of cancer stem cells: a clinical update. Nat Rev Clin Oncol.

[61] Totaro A, Castellan M, Battilana G, Zanconato F, Azzolin L, Giulitti S, Cordenonsi M, Piccolo S (2017). YAP/TAZ link cell mechanics to Notch signalling to control epidermal stem cell fate. Nat Commun.

[62] Totaro A, Castellan M, Di Biagio D, Piccolo S (2018). Crosstalk between YAP/TAZ and notch signaling. Trends Cell Biol.

[63] Kim JH, Park SY, Jun Y, Kim JY, Nam JS (2017). Roles of wnt target genes in the journey of cancer stem cells. Int J Mol Sci.

[64] Gaston-Massuet C, Andoniadou CL, Signore M, Jayakody SA, Charolidi N, Kyeyune R, Vernay B, Jacques TS, Taketo MM, Le Tissier P (2011). Increased Wingless (Wnt) signaling in pituitary progenitor/stem cells gives rise to pituitary tumors in mice and humans. Proc Natl Acad Sci U S A.

[65] Le Grand F, Jones AE, Seale V, Scimè A, Rudnicki MA (2009). Wnt7a activates the planar cell polarity pathway to drive the symmetric expansion of satellite stem cells. Cell Stem Cell.

[66] Katoh M (2017). Canonical and non-canonical WNT signaling in cancer stem cells and their niches: cellular heterogeneity, omics reprogramming, targeted therapy and tumor plasticity (Review). Int J Oncol.

[67] Eyre R, Alférez DG, Santiago-Gómez A, Spence K, McConnell JC, Hart C, Simões BM, Lefley D, Tulotta C, Storer J (2019). Microenvironmental IL1β promotes breast cancer metastatic colonisation in the bone via activation of Wnt signalling. Nat Commun.

[68] Yang L, Shi P, Zhao G, Xu J, Peng W, Zhang J, Zhang G, Wang X, Dong Z, Chen F (2020). Targeting cancer stem cell pathways for cancer therapy. Signal Transduct Target Ther.

[69] Najafi M, Farhood B, Mortezaee K (2019). Cancer stem cells (CSCs) in cancer progression and therapy. J Cell Physiol.

[70] Zhan T, Rindtorff N, Boutros M (2017). Wnt signaling in cancer. Oncogene.

[71] Taniguchi S, Elhance A, Van Duzer A, Kumar S, Leitenberger JJ, Oshimori N (2020). Tumor-initiating cells establish an IL-33-TGF-β niche signaling loop to promote cancer progression. Science.

[72] Markopoulos GS, Roupakia E, Marcu KB, Kolettas E (2019). Epigenetic regulation of inflammatory cytokine-induced epithelial-to-mesenchymal cell transition and cancer stem cell generation. Cells.

[73] Zhao B, Li L, Guan KL (2010). Hippo signaling at a glance. J Cell Sci.

[74] Talukdar S, Bhoopathi P, Emdad L, Das S, Sarkar D, Fisher PB (2019). Dormancy and cancer stem cells: an enigma for cancer therapeutic targeting. Adv Cancer Res.

[75] Gimple RC, Bhargava S, Dixit D, Rich JN (2019). Glioblastoma stem cells: lessons from the tumor hierarchy in a lethal cancer. Genes Dev.

[76] Li Y, Tang T, Lee HJ, Song K (2021). Selective anti-cancer effects of plasma-activated medium and its high efficacy with cisplatin on hepatocellular carcinoma with cancer stem cell characteristics. Int J Mol Sci..

[77] Lin X, Chen W, Wei F, Xie X (2021). TV-circRGPD6 Nanoparticle suppresses breast cancer stem cell-mediated metastasis via the miR-26b/YAF2 axis. Mol Ther.

[78] Raniszewska A, Vroman H, Dumoulin D, Cornelissen R, Aerts J, Domagała-Kulawik J (2021). PD-L1(+) lung cancer stem cells modify the metastatic lymph-node immunomicroenvironment in nsclc patients. Cancer Immunol Immunother.

[79] Liu C, Billet S, Choudhury D, Cheng R, Haldar S, Fernandez A, Biondi S, Liu Z, Zhou H, Bhowmick NA (2021). Bone marrow mesenchymal stem cells interact with head and neck squamous cell carcinoma cells to promote cancer progression and drug resistance. Neoplasia.

[80] Hagiwara M, Yasumizu Y, Yamashita N, Rajabi H, Fushimi A, Long MD, Li W, Bhattacharya A, Ahmad R, Oya M (2021). MUC1-C activates the BAF (mSWI/SNF) complex in prostate cancer stem cells. Cancer Res.

[81] Hsu MY, Yang MH, Schnegg CI, Hwang S, Ryu B, Alani RM (2017). Notch3 signaling-mediated melanoma-endothelial crosstalk regulates melanoma stem-like cell homeostasis and niche morphogenesis. Lab Invest.

[82] Hayakawa Y, Ariyama H, Stancikova J, Sakitani K, Asfaha S, Renz BW, Dubeykovskaya ZA, Shibata W, Wang H, Westphalen CB (2015). Mist1 expressing gastric stem cells maintain the normal and neoplastic gastric epithelium and are supported by a perivascular stem cell niche. Cancer Cell.

[83] Nimmakayala RK, Leon F, Rachagani S, Rauth S, Nallasamy P, Marimuthu S, Shailendra GK, Chhonker YS, Chugh S, Chirravuri R (2021). Metabolic programming of distinct cancer stem cells promotes metastasis of pancreatic ductal adenocarcinoma. Oncogene.

[84] Fendler A, Bauer D, Busch J, Jung K, Wulf-Goldenberg A, Kunz S, Song K, Myszczyszyn A, Elezkurtaj S, Erguen B (2020). Inhibiting WNT and NOTCH in renal cancer stem cells and the implications for human patients. Nat Commun.

[85] Jain S, Annett SL, Morgan MP, Robson T (2021). The cancer stem cell niche in ovarian cancer and its impact on immune surveillance. Int J Mol Sci..

[86] Yuan Y, Wang L, Ge D, Tan L, Cao B, Fan H, Xue L (2021). Exosomal O-GlcNAc transferase from esophageal carcinoma stem cell promotes cancer immunosuppression through up-regulation of PD-1 in CD8(+) T cells. Cancer Lett.

[87] Sphyris N, Hodder MC, Sansom OJ (2021). Subversion of niche-signalling pathways in colorectal cancer: what makes and breaks the intestinal stem cell. Cancers (Basel).

[88] Singh SR (2013). Gastric cancer stem cells: a novel therapeutic target. Cancer Lett.

[89] Zhou C, Fan N, Liu F, Fang N, Plum PS, Thieme R, Gockel I, Gromnitza S, Hillmer AM, Chon SH (2020). Linking cancer stem cell plasticity to therapeutic resistance-mechanism and novel therapeutic strategies in esophageal cancer. Cells.

[90] Pinho S, Frenette PS (2019). Haematopoietic stem cell activity and interactions with the niche. Nat Rev Mol Cell Biol.

[91] Colombo F, Baldan F, Mazzucchelli S, Martin-Padura I, Marighetti P, Cattaneo A, Foglieni B, Spreafico M, Guerneri S, Baccarin M (2011). Evidence of distinct tumour-propagating cell populations with different properties in primary human hepatocellular carcinoma. PLoS ONE.

[92] Wilson GS, Hu Z, Duan W, Tian A, Wang XM, McLeod D, Lam V, George J, Qiao L (2013). Efficacy of using cancer stem cell markers in isolating and characterizing liver cancer stem cells. Stem Cells Dev.

[93] Nakamura T, Sakai K, Nakamura T, Matsumoto K (2011). Hepatocyte growth factor twenty years on: much more than a growth factor. J Gastroenterol Hepatol.

[94] Lau CK, Yang ZF, Ho DW, Ng MN, Yeoh GC, Poon RT, Fan ST (2009). An Akt/hypoxia-inducible factor-1alpha/platelet-derived growth factor-BB autocrine loop mediates hypoxia-induced chemoresistance in liver cancer cells and tumorigenic hepatic progenitor cells. Clin Cancer Res.

[95] Wang X, Sun W, Shen W, Xia M, Chen C, Xiang D, Ning B, Cui X, Li H, Li X (2016). Long non-coding RNA DILC regulates liver cancer stem cells via IL-6/STAT3 axis. J Hepatol.

[96] Fangmann L, Teller S, Stupakov P, Friess H, Ceyhan GO, Demir IE (2018). 3D cancer migration assay with schwann cells. Methods Mol Biol.

[97] Dong HH, Xiang S, Liang HF, Li CH, Zhang ZW, Chen XP (2013). The niche of hepatic cancer stem cell and cancer recurrence. Med Hypotheses.

[98] Houghton J, Stoicov C, Nomura S, Rogers AB, Carlson J, Li H, Cai X, Fox JG, Goldenring JR, Wang TC (2004). Gastric cancer originating from bone marrow-derived cells. Science.

[99] Jiang J, Zhang Y, Chuai S, Wang Z, Zheng D, Xu F, Zhang Y, Li C, Liang Y, Chen Z (2012). Trastuzumab (herceptin) targets gastric cancer stem cells characterized by CD90 phenotype. Oncogene.

[100] Zhi QM, Chen XH, Ji J, Zhang JN, Li JF, Cai Q, Liu BY, Gu QL, Zhu ZG, Yu YY (2011). Salinomycin can effectively kill ALDH(high) stem-like cells on gastric cancer. Biomed Pharmacother.

[101] Proia TA, Keller PJ, Gupta PB, Klebba I, Jones AD, Sedic M, Gilmore H, Tung N, Naber SP, Schnitt S (2011). Genetic predisposition directs breast cancer phenotype by dictating progenitor cell fate. Cell Stem Cell.

[102] Wu ZQ, Li XY, Hu CY, Ford M, Kleer CG, Weiss SJ (2012). Canonical Wnt signaling regulates slug activity and links epithelial-mesenchymal transition with epigenetic breast Cancer 1, early onset (BRCA1) repression. Proc Natl Acad Sci USA.

[103] Tiwari N, Tiwari VK, Waldmeier L, Balwierz PJ, Arnold P, Pachkov M, Meyer-Schaller N, Schübeler D, van Nimwegen E, Christofori G (2013). Sox4 is a master regulator of epithelial-mesenchymal transition by controlling Ezh2 expression and epigenetic reprogramming. Cancer Cell.

[104] Chang CJ, Yang JY, Xia W, Chen CT, Xie X, Chao CH, Woodward WA, Hsu JM, Hortobagyi GN, Hung MC (2011). EZH2 promotes expansion of breast tumor initiating cells through activation of RAF1-β-catenin signaling. Cancer Cell.

[105] Atkinson RL, Zhang M, Diagaradjane P, Peddibhotla S, Contreras A, Hilsenbeck SG, Woodward WA, Krishnan S, Chang JC, Rosen JM (2010). Thermal enhancement with optically activated gold nanoshells sensitizes breast cancer stem cells to radiation therapy. Sci Transl Med.

[106] Lapidot T, Sirard C, Vormoor J, Murdoch B, Hoang T, Caceres-Cortes J, Minden M, Paterson B, Caligiuri MA, Dick JE (1994). A cell initiating human acute myeloid leukaemia after transplantation into SCID mice. Nature.

[107] Park D, Sykes DB, Scadden DT (2012). The hematopoietic stem cell niche. Front Biosci (Landmark Ed).

[108] Krause DS, Scadden DT, Preffer FI (2013). The hematopoietic stem cell niche–home for friend and foe?. Cytom B Clin Cytom.

[109] Colmone A, Amorim M, Pontier AL, Wang S, Jablonski E, Sipkins DA (2008). Leukemic cells create bone marrow niches that disrupt the behavior of normal hematopoietic progenitor cells. Science.

[110] Chirgwin JM (2012). The stem cell niche as a pharmaceutical target for prevention of skeletal metastases. Anticancer Agents Med Chem.

[111] Idowu T, Samadder P, Arthur G, Schweizer F (2017). Amphiphilic modulation of glycosylated antitumor ether lipids results in a potent triamino scaffold against epithelial cancer Cell lines and BT474 cancer stem cells. J Med Chem.

[112] Cazet AS, Hui MN, Elsworth BL, Wu SZ, Roden D, Chan CL, Skhinas JN, Collot R, Yang J, Harvey K (2018). Targeting stromal remodeling and cancer stem cell plasticity overcomes chemoresistance in triple negative breast cancer. Nat Commun.

[113] O'Leary DP, O'Leary E, Foley N, Cotter TG, Wang JH, Redmond HP (2016). Effects of surgery on the cancer stem cell niche. Eur J Surg Oncol.

[114] Lee KM, Giltnane JM, Balko JM, Schwarz LJ, Guerrero-Zotano AL, Hutchinson KE, Nixon MJ, Estrada MV, Sánchez V, Sanders ME (2017). MYC and MCL1 cooperatively promote chemotherapy-resistant breast cancer stem cells via regulation of mitochondrial oxidative phosphorylation. Cell Metab.

[115] Steinbichler TB, Dudás J, Skvortsov S, Ganswindt U, Riechelmann H, Skvortsova II (2018). Therapy resistance mediated by cancer stem cells. Semin Cancer Biol.

[116] Najafi M, Mortezaee K, Majidpoor J (2019). Cancer stem cell (CSC) resistance drivers. Life Sci.

[117] Galle E, Thienpont B, Cappuyns S, Venken T, Busschaert P, Van Haele M, Van Cutsem E, Roskams T, van Pelt J, Verslype C (2020). DNA methylation-driven EMT is a common mechanism of resistance to various therapeutic agents in cancer. Clin Epigenetics.

[118] Xu J, Liu D, Niu H, Zhu G, Xu Y, Ye D, Li J, Zhang Q (2017). Resveratrol reverses doxorubicin resistance by inhibiting epithelial-mesenchymal transition (EMT) through modulating PTEN/Akt signaling pathway in gastric cancer. J Exp Clin Cancer Res.

[119] Jin H, He Y, Zhao P, Hu Y, Tao J, Chen J, Huang Y (2019). Targeting lipid metabolism to overcome EMT-associated drug resistance via integrin β3/FAK pathway and tumor-associated macrophage repolarization using legumain-activatable delivery. Theranostics.

[120] Farmer P, Bonnefoi H, Anderle P, Cameron D, Wirapati P, Becette V, André S, Piccart M, Campone M, Brain E (2009). A stroma-related gene signature predicts resistance to neoadjuvant chemotherapy in breast cancer. Nat Med.

[121] Ghobrial IM, Liu CJ, Zavidij O, Azab AK, Baz R, Laubach JP, Mishima Y, Armand P, Munshi NC, Basile F (2019). Phase I/II trial of the CXCR4 inhibitor plerixafor in combination with bortezomib as a chemosensitization strategy in relapsed/refractory multiple myeloma. Am J Hematol.

[122] Fischer KR, Durrans A, Lee S, Sheng J, Li F, Wong ST, Choi H, El Rayes T, Ryu S, Troeger J (2015). Epithelial-to-mesenchymal transition is not required for lung metastasis but contributes to chemoresistance. Nature.

[123] David CJ, Huang YH, Chen M, Su J, Zou Y, Bardeesy N, Iacobuzio-Donahue CA, Massagué J (2016). TGF-β tumor suppression through a lethal EMT. Cell.

[124] Hua W, Ten Dijke P, Kostidis S, Giera M, Hornsveld M (2020). TGFβ-induced metabolic reprogramming during epithelial-to-mesenchymal transition in cancer. Cell Mol Life Sci.

[125] Calon A, Espinet E, Palomo-Ponce S, Tauriello DV, Iglesias M, Céspedes MV, Sevillano M, Nadal C, Jung P, Zhang XH (2012). Dependency of colorectal cancer on a TGF-β-driven program in stromal cells for metastasis initiation. Cancer Cell.

[126] Ahmadi A, Najafi M, Farhood B, Mortezaee K (2019). Transforming growth factor-β signaling: tumorigenesis and targeting for cancer therapy. J Cell Physiol.

[127] den Hollander MW, Bensch F, Glaudemans AW, Oude Munnink TH, Enting RH, den Dunnen WF, Heesters MA, Kruyt FA, Lub-de Hooge MN, Cees de Groot J (2015). TGF-β antibody uptake in recurrent high-grade glioma imaged with 89Zr-fresolimumab PET. J Nucl Med.

[128] Trachtman H, Fervenza FC, Gipson DS, Heering P, Jayne DR, Peters H, Rota S, Remuzzi G, Rump LC, Sellin LK (2011). A phase 1, single-dose study of fresolimumab, an anti-TGF-β antibody, in treatment-resistant primary focal segmental glomerulosclerosis. Kidney Int.

[129] Lacouture ME, Morris JC, Lawrence DP, Tan AR, Olencki TE, Shapiro GI, Dezube BJ, Berzofsky JA, Hsu FJ, Guitart J (2015). Cutaneous keratoacanthomas/squamous cell carcinomas associated with neutralization of transforming growth factor β by the monoclonal antibody fresolimumab (GC1008). Cancer Immunol Immunother.

[130] Formenti SC, Hawtin RE, Dixit N, Evensen E, Lee P, Goldberg JD, Li X, Vanpouille-Box C, Schaue D, McBride WH (2019). Baseline T cell dysfunction by single cell network profiling in metastatic breast cancer patients. J Immunother Cancer.

[131] Shibue T, Weinberg RA (2017). EMT, CSCs, and drug resistance: the mechanistic link and clinical implications. Nat Rev Clin Oncol.

[132] Huang X, Gan G, Wang X, Xu T, Xie W (2019). The HGF-MET axis coordinates liver cancer metabolism and autophagy for chemotherapeutic resistance. Autophagy.

[133] Singh A, Settleman J (2010). EMT, cancer stem cells and drug resistance: an emerging axis of evil in the war on cancer. Oncogene.

[134] Armstrong AJ, Freedland SJ, Garcia-Blanco M (2011). Epithelial-mesenchymal transition in prostate cancer: providing new targets for therapy. Asian J Androl.

[135] Sun X, Lv X, Yan Y, Zhao Y, Ma R, He M, Wei M (2020). Hypoxia-mediated cancer stem cell resistance and targeted therapy. Biomed Pharmacother.

[136] Martí-Díaz R, Montenegro MF, Cabezas-Herrera J, Goding CR, Rodríguez-López JN, Sánchez-Del-Campo L (2020). Acriflavine, a potent Inhibitor of HIF-1α, disturbs glucose metabolism and suppresses ATF4-protective pathways in melanoma under non-hypoxic conditions. Cancers (Basel).

[137] Min S, Wang X, Du Q, Gong H, Yang Y, Wang T, Wu N, Liu X, Li W, Zhao C (2020). Chetomin, a Hsp90/HIF1α pathway inhibitor, effectively targets lung cancer stem cells and non-stem cells. Cancer Biol Ther.

[138] Soni S, Padwad YS (2017). HIF-1 in cancer therapy: two decade long story of a transcription factor. Acta Oncol.

[139] He M, Wu H, Jiang Q, Liu Y, Han L, Yan Y, Wei B, Liu F, Deng X, Chen H (2019). Hypoxia-inducible factor-2α directly promotes BCRP expression and mediates the resistance of ovarian cancer stem cells to adriamycin. Mol Oncol.

[140] Pinzón-Daza ML, Cuellar-Saenz Y, Nualart F, Ondo-Mendez A, Del Riesgo L, Castillo-Rivera F, Garzón R (2017). Oxidative stress promotes doxorubicin-induced Pgp and BCRP expression in colon cancer cells under hypoxic conditions. J Cell Biochem.

[141] To KK, Poon DC, Wei Y, Wang F, Lin G, Fu LW (2015). Vatalanib sensitizes ABCB1 and ABCG2-overexpressing multidrug resistant colon cancer cells to chemotherapy under hypoxia. Biochem Pharmacol.

[142] Pandya K, Meeke K, Clementz AG, Rogowski A, Roberts J, Miele L, Albain KS, Osipo C (2011). Targeting both Notch and ErbB-2 signalling pathways is required for prevention of ErbB-2-positive breast tumour recurrence. Br J Cancer.

[143] Schott AF, Landis MD, Dontu G, Griffith KA, Layman RM, Krop I, Paskett LA, Wong H, Dobrolecki LE, Lewis MT (2013). Preclinical and clinical studies of gamma secretase inhibitors with docetaxel on human breast tumors. Clin Cancer Res.

[144] Jimeno A, Gordon M, Chugh R, Messersmith W, Mendelson D, Dupont J, Stagg R, Kapoun AM, Xu L, Uttamsingh S (2017). A first-in-human phase I study of the anticancer stem cell agent ipafricept (OMP-54F28), a decoy receptor for wnt ligands, in patients with advanced solid tumors. Clin Cancer Res.

[145] Basset-Séguin N, Hauschild A, Kunstfeld R, Grob J, Dréno B, Mortier L, Ascierto PA, Licitra L, Dutriaux C, Thomas L (2017). Vismodegib in patients with advanced basal cell carcinoma: primary analysis of STEVIE, an international, open-label trial. Eur J Cancer.

[146] Antonarakis ES, Heath EI, Smith DC, Rathkopf D, Blackford AL, Danila DC, King S, Frost A, Ajiboye AS, Zhao M (2013). Repurposing itraconazole as a treatment for advanced prostate cancer: a noncomparative randomized phase II trial in men with metastatic castration-resistant prostate cancer. Oncologist.

[147] Bendell J, Andre V, Ho A, Kudchadkar R, Migden M, Infante J, Tiu RV, Pitou C, Tucker T, Brail L (2018). Phase I study of LY2940680, a smo antagonist, in patients with advanced cancer including treatment-naïve and previously treated basal cell carcinoma. Clin Cancer Res.

[148] Dianat-Moghadam H, Mahari A, Salahlou R, Khalili M, Azizi M, Sadeghzadeh H (2022). Immune evader cancer stem cells direct the perspective approaches to cancer immunotherapy. Stem Cell Res Ther.

[149] Walcher L, Kistenmacher AK, Suo H, Kitte R, Dluczek S, Strauß A, Blaudszun AR, Yevsa T, Fricke S, Kossatz-Boehlert U (2020). Cancer stem cells-origins and biomarkers: perspectives for targeted personalized therapies. Front Immunol.

[150] Ding Z, Li Q, Zhang R, Xie L, Shu Y, Gao S, Wang P, Su X, Qin Y, Wang Y (2021). Personalized neoantigen pulsed dendritic cell vaccine for advanced lung cancer. Signal Transduct Target Ther.

[151] Ying Z, Huang XF, Xiang X, Liu Y, Kang X, Song Y, Guo X, Liu H, Ding N, Zhang T (2019). A safe and potent anti-CD19 CAR T cell therapy. Nat Med.

[152] Singh N, Frey NV, Engels B, Barrett DM, Shestova O, Ravikumar P, Cummins KD, Lee YG, Pajarillo R, Chun I (2021). Antigen-independent activation enhances the efficacy of 4–1BB-costimulated CD22 CAR T cells. Nat Med.

[153] Fathi E, Farahzadi R, Sheervalilou R, Sanaat Z, Vietor I (2020). A general view of CD33(+) leukemic stem cells and CAR-T cells as interesting targets in acute myeloblatsic leukemia therapy. Blood Res.

[154] Wang QS, Wang Y, Lv HY, Han QW, Fan H, Guo B, Wang LL, Han WD (2015). Treatment of CD33-directed chimeric antigen receptor-modified T cells in one patient with relapsed and refractory acute myeloid leukemia. Mol Ther.

